# Exploring Whether Addictions Counselors Recommend That Their Patients Use Websites, Smartphone Apps, or Other Digital Health Tools to Help Them in Their Recovery: Web-Based Survey

**DOI:** 10.2196/37008

**Published:** 2022-06-20

**Authors:** Tyler B Wray

**Affiliations:** 1 Department of Behavioral and Social Sciences Brown University School of Public Health Providence, RI United States

**Keywords:** addiction, alcohol, drug use, substance use, adoption, smartphone, mobile health, mHealth, marketing, dissemination, counselor, health care professional, digital health, eHealth

## Abstract

**Background:**

Hundreds of smartphone apps or websites claiming to help those with addictions are available, but few have been tested for efficacy in changing clinically relevant addictions outcomes. Although most of these products are designed for self-facilitation by users struggling with addictions, counselors and other addictions treatment providers will likely play a critical role in facilitating adoption by integrating their use into counseling or recommending them to their patients. Yet, few studies have explored the practices of addictions counselors in using or recommending addictions-focused digital health tools in their work.

**Objective:**

The aim of this study was to understand whether addiction counselors are recommending that their patients use addictions-focused apps to help them in their recovery, and the factors that affect their desire to do so.

**Methods:**

Licensed addiction counselors practicing in the United States (N=112) were recruited from professional and scientific organizations of alcohol or drug counselors to complete a web-based survey.

**Results:**

In total, 74% (83/112) of counselors had recommended that their patients use a website or smartphone app to assist them in recovery, and those that had done so reported recommending an app with an average of 54% of their patients. The most commonly recommended app or website was SMARTRecovery.org (9%), I am Sober (8%), In the Rooms (7%), Insight Timer (4%), Calm (4%), Sober Tool (4%), Recovery Box (3%), and Sober Grid (3%). The most important reason that counselors recommended the websites or apps was that colleagues or patients told them they found it helpful (55%), followed by their workplaces recommending it (20%) and professional organizations recommending it (10%). Counselors’ intentions to recommend a hypothetical app were strongest for apps that had been tested in rigorous, scientific studies that showed they helped users stay sober or reduce their substance use; 94% (105/112) reported that they would “definitely” or “probably” use such an app.

**Conclusions:**

Most addictions counselors surveyed are already recommending that their patients use apps or websites to help them in their recovery, despite the paucity of available products that have evidence supporting their efficacy for addictions outcomes. One way that product developers could increase adoption among addictions treatment providers is to make efficacy testing a priority and to disseminate results through professional organizations and clinics.

## Introduction

The market of health-related digital tools, such as websites and smartphone apps, is continuing to grow exponentially, with more than 90,000 health apps added to major app stores in 2020 [1]. Total funding for digital health ventures also surpassed US $21.3 billion in the first three-quarters of 2021, up from US $1.1 billion just a decade ago [2]. Mental health–focused products make up the largest share of apps available to help manage health conditions [1], and several mental wellness apps (eg, Calm and Headspace) were represented among the most downloaded apps in 2020 [3].

Although hundreds of addictions-focused apps are currently available online and in app stores, only a handful of these products have been formally tested for their efficacy or effectiveness in changing addiction-relevant outcomes such as alcohol or drug use and problems or substance use disorder symptoms or severity [4]. Similarly, only a few addictions-focused apps have sought premarket clearances or approvals that require them to submit data on their products’ safety or efficacy from controlled studies [1]. This means that evidence supporting clinical benefit is only available to the public for a very small number of addictions-focused apps. However, a number of such products have been developed and have shown promising effects on a variety of addictions outcomes [5]. A handful of products have also recently sought Food and Drug Administration approval or clearance for their addictions-focused products to begin marketing their benefits for those in recovery (eg, reSET [6]). Although no treatment guidelines or major professional organizations have yet explicitly suggested that clinicians use or recommend any such products, it is very likely that one or more of these products will prove beneficial enough to achieve this degree of support at some point in the near future.

Most of the addiction-focused websites and smartphone apps that have been developed thus far have been primarily designed for self-facilitation, meaning that patients are primarily intended to use them on their own, privately [7]. For this reason, addiction-focused products, such as other mental health–focused apps, often pursue at least some patient-focused marketing strategies as a part of their overall marketing plans. These strategies, such as direct-to-consumer advertising, would likely be more successful if they highlighted any benefits their products have shown on addictions outcomes in controlled research. That is, conducting research evaluating the efficacy of these apps on clinically relevant outcomes (such as alcohol or drug use and problems or substance use disorder symptoms) may be important for increasing confidence and interest in the product among consumers. However, the uptake of these products among patients may also be higher when treatment providers recommend that they use them [8], leading many product developers in other spaces to pursue marketing their consumer-focused digital health products through medical and mental health providers. Some addiction-focused products have also pursued regulatory clearance or approval under a prescription-based model (eg, reSET [6]), meaning that patient uptake is not possible at all without a provider’s recommendation. Other products, such as virtual reality experiences [9], may also be explicitly intended to be used collaboratively by counselors and patients during treatment sessions, which is another scenario that requires counselor adoption in order for the product to be used by patients. Given these approaches, understanding the practices of addictions providers in recommending websites or apps to their patients and factors affecting their likelihood of recommending them is critical for successfully disseminating addiction-focused digital health products. Few such studies have been conducted to date.

In this study, I explored whether licensed, practicing addictions counselors are currently recommending that their patients use websites or smartphone apps to help them in their recovery, and examined some factors that could affect their desire to do so. I also examined marketing or dissemination content and venues that could be most likely to reach and encourage addiction counselors to recommend websites or apps to their patients.

## Methods

### Participants

Participants (N=112) were recruited from email groups, listserv postings, and advertisements in newsletters maintained by professional and scientific organizations of drug or alcohol counselors, as well as social media posts and advertisements, to participate in a web-based survey from February to July 2021. Eligible participants (1) were at least 18 years old, (2) were able to read fluently in English, (3) were licensed to provide counseling in the United States, and (4) provided alcohol or drug counseling for at least 15% of their typical work week. Moreover, another manuscript that is currently in review reported on other data from this survey (TB Wray, unpublished data, September 2021).

### Procedures

The participants first completed a web-based screening survey to determine eligibility. If the respondents were eligible and interested, they were asked to provide informed consent and contact information before being redirected to the full survey. The main survey took participants an average of about 25 minutes to complete. Those participants who completed the full survey were compensated with a US $20 gift card, sent via email.

### Ethics Approval

All procedures were reviewed by the Brown University IRB and were determined to be exempt from ongoing review and approval (protocol # 2101002892), because the study procedures only involved a single survey that collected data on a topic that, if these data were inadvertently disclosed, would not reasonably place the subjects at risk of criminal or civil liability or damage their financial standing, employability, educational advancement, or reputation.

### Measures

Since few validated measures have been developed to assess counselors’ perceptions about digital health apps, most items were created for this study. The question about what websites or app counselors had recommended allowed respondents to enter text freely. To evaluate whether each product had been the focus of published, peer-reviewed research, I searched several databases (eg, Google Scholar, PubMed, and Food and Drug Administration’s Premarket Approval 510k databases, if applicable) for each by their listed brand name, as well as websites that the product or its developer maintained for relevant research reports. Questions about counselors’ *perceptions of their patients’ use* of addictions-focused apps were assessed on a 1 (*not at all*) to 5 (*a lot*) scale. Questions asking counselors to estimate the *percentage of their patients they recommended apps* *to* were rated on a slider scale from 1% to 100%. Items asking the counselors to rate their intentions to recommend a hypothetical app under various conditions (eg, if rigorous, scientific studies had shown it was effective) were rated on a 1 (*not at all*) to 5 (*definitely yes*) scale. The question asking about the counselors’ most important reservations about recommending digital health products to their patients displayed 9 potential reservations (e., “I don’t know any that help with that, they would probably cost too much”) plus an “other” option with free text entry. The participants were instructed to select those reservations that were concerns for them, and then rank them from most to least important. This item was displayed only to participants who had not ever recommended an app or website and expressed at least some reservation about doing so in the future (ie, responded lower than “definitely yes” on the item asking about their intentions to recommend any app or website to their patients in the future; N=26).

### Data Analysis Plan

Data from all complete responses for the items reported were included in these analyses. Descriptive statistics were calculated for all demographic and professional characteristics. Basic summary statistics were used (percentages, medians, and interquartile range for Likert scale data) for all focal study items. All analyses were conducted in Stata 16 (StataCorp).

## Results

Participant demographic characteristics are presented in Table 1, and professional characteristics are presented in Table 2. The respondents (N=112) were largely master-level clinicians (n=72, 64.3%). Nearly half (n=52, 46.4%) were licensed substance abuse counselors, with many social workers (n=29, 25.9%) and mental health counselors (n=20, 17.9%) as well. Most participants reported working in an outpatient addiction treatment center, in private practice, outpatient mental health centers, or criminal justice settings. According to national data on the substance use disorder treatment workforce [10,11], a higher percentage of participants in this study were counseling staff (99% vs 42%), had a graduate education (75% vs 57%), and earned a higher annual salary (US $71,937 vs US $48,520) compared to the average substance use disorder treatment professional in the United States.

**Table 1:** Demographic characteristics of the participants (N=112).

Characteristics		Values
Age (years), mean (SD; range)		45.6 (12.3; 25-76)
Sex (female)		86 (76.8)
**Race or ethnicity, n (%)**		
	White	93 (83.0)
	Black or African American	13 (11.6)
	Asian	2 (1.8)
	American Indian or Alaska Native	0 (0)
	Multiracial	4 (3.6)
	Hispanic or Latino	5 (4.5)
**Sexual orientation, n (%)**		
	Heterosexual or straight	96 (85.7)
	Gay or lesbian	7 (6.3)
	Bisexual	7 (6.3)
	Other or chose not to respond	2 (1.8)
**US region of primary residence, n (%)**		
	Northeast	55 (49.1)
	South	36 (32.1)
	Midwest	13 (11.6)
	West	8 (7.1)

**Table 2:** Professional characteristics of the participants (N=112).

Characteristics		Values
**Education, n (%)**		
	Some college	1 (0.9)
	Bachelor’s degree	22 (19.6)
	Some grad school	5 (4.5)
	Master’s degree	72 (64.3)
	Some doctoral work	3 (3.0)
	Doctorate degree (PhD, PsyD, DNP)	8 (7.1)
	Medical doctorate (MD, DO)	1 (0.9)
Annual income (US $), mean (SD)		71,937 (23,618)
Employed full-time, n (%)		104 (92.9)
**Professional discipline, n (%)**		
	Psychologist	6 (5.4)
	Social worker	29 (25.9)
	Substance abuse counselor	52 (46.4)
	Health educator	2 (1.8)
	Mental health counselor	20 (17.9)
	School counselor	1 (0.9)
	Physician (other than psychiatry)	1 (0.9)
	Other	1 (0.9)
Years of training in addictions counseling, mean (SD)		3.6 (2.1)
Years of experience in addictions counseling, mean (SD)		10.4 (6.4)
Number of sessions with each addictions client, mean (SD)		14 (8.8)
**Primary work setting, n (%)**		
	Community hospital	4 (3.6)
	Academic hospital	6 (5.4)
	Veterans’ affairs or military hospital	5 (4.5)
	Outpatient mental health center	16 (14.3)
	Inpatient mental health center	5 (4.5)
	Outpatient addiction treatment center	21 (18.8)
	Inpatient addiction treatment center	4 (3.6)
	Prison or detention center	16 (14.3)
	Community medical clinic or health center	7 (6.3)
	Private medical clinic or health center	1 (0.9)
	Government or social service center	2 (1.8)
	K-12 school	2 (1.8)
	Private practice	19 (16.0)
	Other	2 (1.8)
**Primary counseling approach, n (%)**		
	12-step facilitation	5 (4.5)
	Cognitive behavioral therapy	15 (13.4)
	Motivational interviewing	51 (45.5)
	Acceptance and commitment therapy	7 (6.3)
	Mindfulness-based relapse prevention	3 (2.7)
	Dialectical behavior therapy	2 (1.8)
	Narrative therapy	3 (2.7)
	Harm reduction	2 (1.8)
	Eclectic	8 (7.1)
	Other	16 (14.3)

In total, 74% (83/112) of the respondents had recommended that their patients with addiction use a website or smartphone app to assist them in recovery. Among those who had ever recommended a smartphone app or website, participants reported recommending 122 different apps or websites (Table 3). These participants also reported recommending one of these apps to an average of 54% of their patients. Unsurprisingly, the most recommended category of website or app was general recovery support apps, which provide a wide variety of features and content intended to facilitate recovery, including forums or groups to provide social support; meeting or service locators; daily meditations; and behavior, mood, and thought tracking, among others. One such platform, SMARTRecovery.org (9%, 11/122) was the most recommended application overall. Meditation and mindfulness apps were the second most common type of website or app that counselors recommended to their patients, with Calm (4%, 5/122) and Insight Timer (4%, 5/122) among the most popular of these. Sobriety trackers were the next most recommended category of website or app, and I Am Sober (7%, 9/122) was among the most recommended overall. In the Rooms, which is a 12-step facilitation app that links users with Alcoholics Anonymous and Narcotics Anonymous meetings nearby was also among the most common recommendations overall (7%, 9/122). Finally, some counselors also reported recommending that their patients use websites or apps that provide videos, podcasts, or written text that deliver recovery-related education and inspiration. Among the most popular of these were the Hazelden suite of smartphone apps, such as Twenty-Four Hours a Day, which provides written affirmational or inspirational content to users. Only 1 of the top 10 most frequently recommended websites or apps, Calm, has been the focus of published, peer-reviewed efficacy or effectiveness research exploring the effects of that specific product on outcomes that were potentially related to addictions. None of the apps that the counselors recommended have yet been the focus of efficacy research specifically on addiction outcomes, such as alcohol or drug use and problems, or substance use disorder symptoms.

The most frequently reported reason that counselors recommended the websites or apps to their patients was that colleagues or patients had told them they found it helpful (55%, 45/82), followed by their places of work recommending it (20%, 16/82) and professional organizations or groups recommending it (10%, 8/82). Only 9% (7/82) of the counselors reported recommending a particular website or app to their patients because rigorous, scientific studies had shown that it was helpful. However, all counselors reported the strongest intentions to recommend that their patients use a website or app to help them in recovery if rigorous, scientific studies (eg, randomized controlled trials) had been conducted on the efficacy of that website or app and had shown that using it helped patients stay sober or reduce their substance use; 94% (105/112) reported that they would “definitely” or “probably” recommend a product that was supported by this degree of evidence. The counselors also had high intentions to recommend websites or apps if others they knew (eg, colleagues or patients) found it helpful; 92% (103/112) reported that they would “definitely” or “probably” recommend such a product. Finally, only 38% (43/112) of the counselors reported that they would “definitely” or “probably” recommend an app or website to patients if its developers claimed it was helpful for users in recovery.

Of counselors who had recommended at least one app (82/112, 74%), the most common way they heard about the apps they recommended was web-based search, followed by other counselors or clinicians, patients, and professional organization conferences or conventions. By contrast, most participants reported that they would *like* to hear about addiction-focused apps through other counselors or clinicians and patients, followed by hearing about them through conferences or publications (eg, newsletters) maintained by their professional organizations, or through seminars at work (Table 4).

Of the 30 counselors who had not ever recommended a website or app, 79% (n=24) said they would either “probably” or “definitely” recommend that their patients use a website or smartphone app to help their patients stay sober or reduce their alcohol or drug use. However, the most frequently chosen reservation among those who had not recommended an app or were uncertain about doing so was that they did not know of any app that had been shown scientifically to help with addictions (11.6%). Other frequently identified and highly ranked reservations were concerns that their patients would not actually use them, not being generally aware of any websites or apps, and believing that their patients do not have the resources to use a computer or a smartphone (Table 5).

**Table 3:** Apps and websites that addiction counselors most recommended to their patients in recovery and their core functions (only categories with >1 recommendation are represented).

App or website category	Description^a^	Percent^b^	Most common sites or apps^b^ (%)
General recovery support	Provides several features, such as forums, meeting or service locators, daily meditations, sobriety counters, and calculators, intended to provide general recovery support.	21.3	SMARTRecovery.org (9) RecoveryBox (2) Sober Grid (2)
Meditation or mindfulness	Provides content to help users develop mindfulness skills, such as guided meditations, education, and badges or rewards.	19.7	Calm (4) Insight Timer (4) Headspace (2)
Sobriety trackers	Primarily helps users track their length of sobriety, including various calculators of benefit (eg, money saved)	18.0	I Am Sober (7) Sober Tool (4) Nomo (2)
12-step facilitation	Helps connect users to AA^c^ or NA^d^ meetings or hosts meetings on their platforms and other AA- or NA-associated content.	14.8	In the Rooms (7) Meeting Guide (1) 12 Step Companion (1)
Inspiration, podcasts, or lectures	Provides video, audio, or text on recovery-related topics primarily to educate or motivate (inspire) users.	7.4	Hazelden Recovery Apps (4) SoberCast (2) YouTube (2)
Social media	General social media sites or apps; counselors recommended specific recovery communities and groups.	7.4	Facebook (2) Reddit (2)
General mental health	Provides content intended to support general mental health, such as mood tracking and thought diaries.	2.5	CBT Thought Diary (1) Mood Meter (1) Woebot (1)
Smoking cessation	Provides support for smoking cessation, such as quit planning, nicotine replacement therapy, and social support.	2.5	smokefree.org (1) NY Quits (1) QuitlineNC (1)
Unknown or other	App or website did not fit into other categories, was not found, or its purpose was unclear.	4.9	Strengths Finder (1) Weconnect (1)

^a^Apps or websites were grouped according to their general purposes. Many apps or websites across categories contain similar sets of features (eg, social support and daily meditations).

^b^All percentages represent the percent of all identified apps or websites that the participants reported recommending.

^c^AA: Alcoholics Anonymous.

^d^NA: Narcotics Anonymous.

**Table 4:** The platform from which counselors first heard of the addiction-focused apps they recommended, and where they would like to hear about them^a^.

Platform or person	*Did* hear (N=82), frequency (%)	*Would like* to hear (N=112), frequency (%)
Web-based search	39 (34.8)	27 (24.1)
Other counselors or clinicians	36 (32.1)	75 (67.0)
Other patients or patients	23 (20.5)	49 (43.8)
Professional organization conference or convention	15 (13.4)	38 (33.9)
Professional organization newsletter or bulletin	14 (12.5)	44 (39.3)
App store (Apple App Store or Google Play)	12 (10.7)	11 (9.8)
Workshop or seminar at work	11 (9.8)	34 (30.4)
Other	7 (6.3)	0 (0)
Scholarly journal article	3 (2.7)	20 (17.9)
TV advertisement	1 (0.9)	6 (5.4)
Internet advertisement	0 (0)	10 (8.9)
Company press release	0 (0)	0 (0)

^a^Participants could select multiple ways in which they heard about the apps or websites they recommended. These data represent the number of times each method was identified and the percentage of all responses in which that method was selected.

**Table 5:** Addiction counselors’ most frequently chosen reservations about recommending that their patients use websites or apps to help them with their recovery^a^ (N=26).

Comments	Frequency^b^ (%)	Rank, median (IQR)
I don’t know of any websites or apps that help with addictions.	9 (8.0)	2 (1;3)
I don’t know of any that have been shown scientifically to help with addictions.	13 (11.6)	2 (1;2)
I don’t think websites or apps can help people reduce their alcohol or drug use.	5 (4.5)	4 (2;7)
They would probably cost too much.	11 (9.82)	2 (2;3)
I don’t think my patients would want to use them.	7 (6.3)	3 (2;4)
I don’t think my patients would actually use it even if they wanted to.	10 (10.7)	2 (1;3)
I don’t think they have the resources (a computer or smartphone) to use them.	9 (8.0)	1 (1;2)
I’m concerned it may do more harm than good	7 (6.3)	3 (2;5)
I’m concerned that, if I did, they may stop coming to counseling.	4 (3.6)	6.5 (3;8.5)
Other	3 (2.7)	3 (3;4)

^a^This item was only rated by respondents who expressed never having recommended an app to a patient in recovery *and* expressed some reservation about doing so in the future (N=26).

^b^The respondents could select multiple reservations. Frequencies reflects the number of times each reason was chosen and the percentage of all selections.

## Discussion

### Principal Findings

The results of this study showed that 3 (75%) of every 4 licensed practicing addictions counselors surveyed had recommended that a client use a website or smartphone app to assist them in their recovery. The results also showed that counselors who had recommended an app did so with about half of their patients, suggesting that many counselors are already actively recommending addictions-focused or mental health–focused digital health products to most of their patients. However, only 1 of the apps that the counselors listed, Calm—a mindfulness and meditation app primarily marketed for improving sleep and reducing stress—has been the focus of peer-reviewed, published efficacy research, which included outcomes that are potentially relevant to addictions to date. Randomized controlled trials have shown that using the Calm app improves sleep outcomes among adults with sleep disturbance [12]. Others have shown that it could reduce depression and anxiety among patients with cancer [13,14], and that it could reduce stress among college students [15]. Although none of these research projects focuses on addictions outcomes specifically, sleep disturbances and mental health challenges frequently co-occur with substance use disorders [16,17], and counselors may be recommending the Calm app to patients to help them with those conditions. None of the apps that the counselors reported recommending to their patients had been the focus of published, peer-reviewed efficacy research focusing on outcomes directly related to addictions. However, one of the most frequently recommended applications, SMARTRecovery.org (Self-Management and Recovery Training), is based on a face-to-face, group-based approach to addictions treatment, and this nondigital treatment has shown some promise in preliminary studies [18]. SMARTRecovery.org connects users with online group meetings and other web-based tools (eg, worksheets and exercises), but the efficacy of these web-based tools on addictions outcomes has not yet been evaluated [19]. Overall, these findings suggest that the desire of counselors is high enough to provide patients who have addiction with tools that can help them in their recovery; counselors may be recommending apps to them despite the limited availability of evidence about whether any of these apps are helpful in changing addictions-focused outcomes, such as alcohol or drug use and problems, or symptoms of substance use disorder. This is further supported by results suggesting that most counselors found the apps they recommended via online search, suggesting that many are actively searching for solutions for their patients on their own. Although the apps that the counselors recommended may be of some benefit for some patients and are unlikely to impair their recovery, doing so is not without risk; patients and counselors may spend money and time on solutions that are not helpful, and doing so could reduce trust in treatment generally. However, these findings also affirm that counselors could play an important role in encouraging the uptake of evidence-based, addictions-focused digital health tools generally.

The findings of this study also suggest that providing a strong evidence base for addictions-focused digital health products could increase the number of counselors who recommend these products to their patients. Across all counselors, intentions to recommend an app or website to their patients were highest if rigorous, scientific studies had shown it was helpful in reducing addictions outcomes. Many developers often consider efficacy testing to be a priority for helping achieve their regulatory goals; however, these results suggest that efficacy testing may also benefit the marketing and dissemination of addictions-focused products by increasing the number of counselors who recommend it to their patients. The results also suggested that counselors’ intentions to recommend were also high for a hypothetical app that their colleagues or patients found helpful, which could suggest the importance of including endorsements and testimonials from trusted sources in marketing materials. This conclusion is further supported by the counselors’ preferences that they hear about addictions-focused apps or websites through colleagues or patients. However, counselors also indicated that they would prefer to hear more about addictions-focused apps or websites from the professional associations they are involved in or their workplaces; this suggested that marketing efforts focused on both professional associations and large care providers could play an important role in successfully marketing addictions-focused digital health products. It could also suggest that implementation approaches that involve steps such as identifying and training local opinion leaders, early adopters, and “champions” of the intervention among counselors in specific professional organizations or treatment centers may be a particularly potent strategy for encouraging the uptake of any future evidence-based addictions-focused website or app [20].

### Limitations

Several limitations are important to note. First, this sample represents a relatively small subset of addictions care providers; therefore, findings could be different in a larger sample or a sample composed of participants from a different variety of professional backgrounds. Second, the study materials (eg, recruitment advertisements and consent documents) disclosed that this study was about the role of technology in addictions counseling (as the institutional review board required it), so this may have attracted participants who are generally interested in using technology in addictions care. Therefore, the study may have overestimated the number of counselors who had or were willing to recommend apps to their patients. Third, the participants in the study were primarily of a White, non-Hispanic racial or ethnic background and were master-level clinicians. Therefore, the results reported here may not be generalizable to addictions counselors with other racial or ethnic backgrounds or education levels. Lastly, all items used in this study were created specifically for this study, so their reliability or validity has not yet been established.

### Conclusions

In conclusion, the results of this study showed that a large majority of licensed, practicing addictions counselors had recommended apps or websites to their patients to help them in their recovery, and that those who had, did so with most of their patients. However, none of the apps that the counselors recommended to their patients had been supported by published, peer-reviewed, scientific research about their impact on addictions outcomes, likely because few such products are broadly available or marketed currently. However, the findings also showed that intentions to recommend a hypothetical addictions-focused digital health product were highest when rigorous research was available showing that the product helped reduce clinically relevant addiction outcomes, which in turn suggests that conducting this research could encourage counselors to recommend these products.
